# Cost-effectiveness analysis of XELOX for metastatic colorectal cancer based on the NO16966 and NO16967 trials

**DOI:** 10.1038/sj.bjc.6605114

**Published:** 2009-06-02

**Authors:** T Shiroiwa, T Fukuda, K Tsutani

**Affiliations:** 1 Department of Drug Policy and Management, Graduate School of Pharmaceutical Sciences, the University of Tokyo, Tokyo 113-0033, Japan; 2 Department of Health Economics and Epidemiology Research, School of Public Health, the University of Tokyo, Tokyo 113-0033, Japan

**Keywords:** cost-effectiveness analysis, capecitabine, colorectal cancer, FOLFOX

## Abstract

**Background:**

The purpose of the study was to evaluate the cost-effectiveness of capecitabine plus oxaliplatin (XELOX) compared with 5-fluorouracil/folinic acid and oxaliplatin (FOLFOX4) as first-line or second-line chemotherapy in patients with metastatic colorectal cancer.

**Methods:**

On the basis of NO16966 and NO16967 trials, mean costs and effectiveness were calculated from patient-level data. Until the disease progressed, the mean costs were calculated from the perspective of health-care payers in Japan. We estimated mean quality-adjusted progression-free survival days (QAPFSD), considering adverse events and patient preference for chemotherapy regimens. Utility scores were obtained by a web-based survey from general people, randomly sampled from a large panel adjusted for sex and age.

**Results:**

Incremental effectiveness of XELOX as first-line and second-line chemotherapy for colorectal cancer patients was significantly greater. By use of XELOX, patients gained 10.5 QAPFSD from first-line treatment or 11.3 QAPFSD from second-line treatment. Capecitabine plus oxaliplatin (XELOX) was also proven to significantly reduce treatment costs by €3000 (JPY 360 000) and €2300 (JPY 270 000) for first-line and second-line treatment, respectively. In health-care settings in the United Kingdom, XELOX decreased medical costs for National Health Service by £7600 and £3900 for patients who received first-line and second-line treatment, respectively.

**Conclusion:**

Capecitabine plus oxaliplatin (XELOX) as first-line and second-line chemotherapy was ‘dominant’. In terms of effectiveness and cost, XELOX was superior to FOLFOX4.

A total of 40 000 people die each year from colorectal cancer, which is the third leading cause of death due to malignant neoplasms after lung cancer and stomach cancer (Ministry of Health, Labour and Welfare, 2007) in Japan. Moreover, the age-adjusted incidence rate of colorectal cancer is approximately 40 per 100 000 people, representing the second highest incidence rate for a malignant neoplasm after stomach cancer (Center for Cancer Control and Information Services, 2007).

Significant progress in chemotherapy of metastatic colorectal cancer (MCRC) has been made in recent years (Kelly and Goldberg, 2005; Meyerhardt and Mayer, 2005). Until approximately the year 2000, 5-fluorouracil (5-FU) plus leucovorin (LV) was the standard regimen used in most countries, but oxaliplatin- or irinotecan-containing regimens were developed rapidly and are now widely administered to many MCRC patients. As a result, median overall survival (OS) of patients with MCRC has improved steadily over this decade (Meyerhardt and Mayer, 2005). As monoclonal antibody drugs, such as bevacizumab or cetuximab, are administered to many of them in addition to chemotherapy, the current challenge is to maintain good quality of life (QOL) and prolong survival for MCRC patients who receive chemotherapy.

Oxaliplatin regimens, FOLFOX4 (de Gramont *et al*, 2000) or FOLFOX6 (Tournigand *et al*, 2004), are the most frequently used chemotherapies for MCRC. Unfortunately, these two regimens frequently yield oxaliplatin-caused adverse events (AEs), especially neuropathy, which greatly influences QOL. Furthermore, patients receiving a FOLFOX regimen must undergo a 2-day continuous infusion of 5-FU every 2 weeks. Even without AEs from the chemotherapy, the 2-day continuous infusion may decrease patient QOL. To avoid continuous infusion, as is necessary for 5-FU administration, the use of oral fluorinated pyrimidine drugs, such as capecitabine, has recently increased.

Capecitabine belongs to the fluorinated pyrimidine class of anticancer drugs. It is metabolised in the body and eventually converted into FU within tumour tissue, where it shows antitumour activity (Miwa *et al*, 1998). Because the enzyme (thymidine phosphorylase) responsible for the last conversion step is more concentrated in tumour tissue than in normal tissues, FU levels in tumour tissue are selectively increased (Schuller *et al*, 2000). Thus, capecitabine offers an improved tolerability profile compared with FU/LV with respect to some systemic AEs (Cassidy *et al*, 2002). Also, as capecitabine is given orally, it avoids the need for intravenous drug preparation and administration and associated visits to the clinic. Patient preference data also suggest that patients generally prefer oral over intravenous therapy (Twelves *et al*, 2006).

Capecitabine plus oxaliplatin (XELOX) (Hochster *et al*, 2006; Diaz-Rubio *et al*, 2007; Porschen *et al*, 2007; Rothenberg *et al*, 2008; Saltz *et al*, 2008) is an improved regimen which includes capecitabine but does not require 5-FU infusion. NO16966 (Saltz *et al*, 2008) and NO16967 (Rothenberg *et al*, 2008) clinical trials, which compared XELOX and FOLFAX4, demonstrated non-inferiority of XELOX as first-line (Saltz *et al*, 2008) and second-line chemotherapy (Rothenberg *et al*, 2008), based on progression-free survival (PFS) (hazard ratio (HR)=1.05; 97.5% confidence interval (CI)=0.94–1.18 in the NO16966 trial) (HR=1.03; 95% CI=0.87–1.24 in the NO16967 trial). However, it is unknown which regimen is more cost-effective.

In many developed countries, increased medical costs represent a major issue. Making decisions about health-care interventions that consider costs, in addition to efficacy and safety, has become increasingly important. Our objective was to compare not only costs but also quality-adjusted life-years (QALY), that is, whether people clearly prefer oral chemotherapy to intravenous chemotherapy considering both gained life year and utility of chemotherapy. This study measured original utility scores, and people’s preference for oral chemotherapy was incorporated into QALY calculations and thus reflected in the analysis. Thus, we investigated the cost-effectiveness of XELOX in comparison with FOLFOX4 from the perspective of health-care payers.

## Materials and methods

Our economic evaluation of XELOX was performed retrospectively, based on patient-level data from two multinational randomised clinical trials, NO16966 and NO16967.

### Patients receiving chemotherapy regimens

In the NO16966 trial of first-line chemotherapy, MCRC patients were randomised to four groups: XELOX (i.v. infusion of oxaliplatin at 130 mg m^−2^ on day 1 every 3 weeks, oral capecitabine at 1000 mg m^−2^ b.i.d. for 2 weeks followed by 1 week without treatment); XELOX+bevacizumab (BV) (i.v. BV at 7.5 mg kg^−1^); FOLFOX4 (i.v. infusion of oxaliplatin at 85 mg m^−2^ on day 1 of a 2-week cycle, i.v. infusion of LV at 200 mg m^−2^ on days 1 and 2, i.v. 5-FU delivered as 400 mg m^−2^ bolus injection on days 1 and 2 followed by a continuous infusion at 600 mg m^−2^ over a period of 22 h on days 1 and 2); and FOLFOX4+BV (i.v. BV at 5 mg kg^−1^). Two BV groups in the NO16966 trial were excluded from the current analysis. In the NO16967 trial of second-line chemotherapy, patients were randomised to XELOX or FOLFOX4 treatment, as there were no BV groups in NO16967 trial.

### Economic evaluation

We performed a cost-effectiveness study of XELOX as first-line and second-line chemotherapy, as compared with FOLFOX4, for MCRC patients. The cost-effectiveness of XELOX for first-line therapy was assessed based on data from the NO16966 trial, whereas the cost-effectiveness of second-line treatment with XELOX was evaluated based on data from the NO16967 trial.

Because the prognoses for both XELOX and FOLFOX4 treatments of MCRC were the same, incremental effectiveness was estimated by the difference in quality-adjusted progression-free survival days (QAPFSD). Hazard ratio of OS was 0.98 (97.5% CI=0.82–1.17) in the NO16966 trial and 1.03 (95% CI=0.87–1.23) in the NO16967 trial. Therefore, the difference in QAPFSD/365 days was thought to be equal to the difference in QALY. As a secondary analysis we also calculated the difference in progression-free survival days (PFSD). For the same reason, this can be regarded as the difference of life years.

Incremental cost was calculated by the difference in total costs during the period of PFS. Because the use and duration of post-treatment chemotherapy of XELOX- or FOLFOX4-treated patients was not considered to be different, the costs during PFS were regarded as the incremental total costs.

In this cost-effective analysis, the perspective was that of health-care payers, which included only direct medical cost, not indirect cost (e.g., work loss). Neither costs nor outcome were discounted because of the short time horizon of the trials.

### Patients

For the analysis set of patients, we used the intent-to-treat (ITT) population, and patients who did not receive even one dose of predetermined protocol chemotherapy were excluded. From the NO16966 trial, the ITT population used in our analysis was XELOX (*n*=655) and FOLFOX4 (*n*=649); from the NO16967 trial, the ITT population used in our analysis was XELOX (*n*=311) and FOLFOX4 (*n*=308). Of the ITT population from the two trials used in our analysis, baseline characteristics of the patients are shown in Table 1.

### Utility scores and outcome measurement

Using time tradeoff (TTO) methods, utility scores for both chemotherapy regimens and seven grade 3/4 AEs were assessed through an online survey of the general population. For cost-effectiveness analysis, the use of utility scores from the general populous rather than that from the patient population is recommended (Gold *et al*, 1996). The seven AEs of febrile neutropenia, nausea/vomiting, diarrhoea, hand–foot syndrome, fatigue, peripheral neuropathy, and stomatitis due to chemotherapy were chosen because they were the main chemotherapy-related AEs observed in the NO16966 trial.

**Figure 1:**
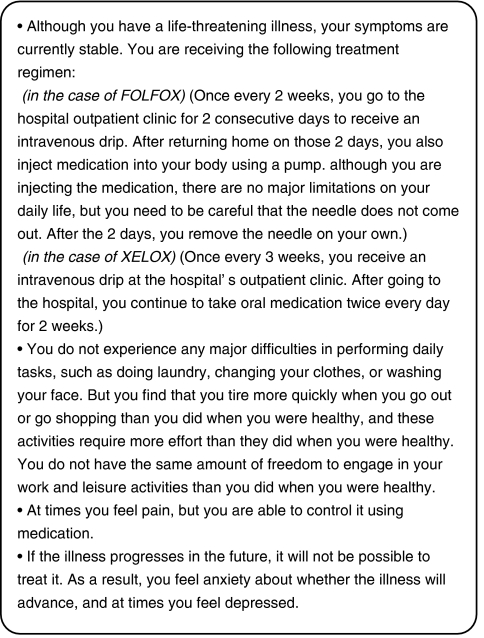
The health state describing an MCRC patient receiving FOLFOX or XELOX.

We developed first draft of health states designed to describe an MCRC patient based on interviews with experts, literature review (Lloyd *et al*, 2006), NCI Common Terminology Criteria for Adverse Events (CTCAE), and health-related quality of life (HRQOL) questionnaires (especially, the EQ-5D questionnaire). First draft of health states were reviewed independently by two experienced oncologists and two oncologic clinical research coordinators. After we received their comments, we improved descriptions of health states and made second draft accordingly. Following review of the second draft on health states by other clinical research coordinators, we carefully modified the descriptions to achieve a completed document on final health states (Figure 1).

Respondents were asked to read about one health state and imagine that they currently live in that state of health. Approximately 180 survey responses of each health state were collected from the largest Internet-based panel in Japan. Population size of the field panel (INTAGE Interactive Inc., Tokyo, Japan) is 760 000 people. Respondents were randomly sampled, stratified by gender and age. The statistical analysis method utilised was described earlier in a report by Lloyd *et al* (2006).

Using measured utility scores, we calculated the mean QAPFSD based on PFS and onset date and end date of grade 3/4 AEs. Mean QAPFSD of each group was estimated by nonparametric, direct methods for patient data (Willan and Briggs, 2007). The CI for mean QAPFSD was constructed by the bootstrap method (Efron and Tibshirani, 1993).

### Medical resource use and costs

Consumption of medical resources on anticancer drugs and premedication drugs before oxaliplatin administration was estimated by patient dose of medications. Outpatient chemotherapy fee (including fees for outpatient chemotherapeutic medications, fee for blood test, fee for diagnostic imaging, and pharmacy fee) was added to medical costs, following the schedule determined by the chemotherapy protocol. Unit costs were calculated for the year 2007 in Japan, based on the reimbursement schedule of social insurance in 2006 (Laboratory, 2006) and the drug tariff in 2007 (Jiho, 2007).

Because marketing of generic levo leucovorin (l-LV) began in 2007, we calculated the cost of FOLFOX4 using both the price of branded and generic l-LV. Censored data were considered to calculate the mean cost per patient, according to the method of Lin *et al* (1997). Also, CIs for mean cost were constructed by the bootstrap method.

Costs of medication for management of treatment-related AEs were also considered. Costs of hospitalisation for AEs were not included in the base-case analysis, as no data on actual resource consumption due to hospitalisation for AEs were available. Impact of costs on the current analysis was determined by sensitivity analyses.

All costs expressed in Japanese Yen (¥) were converted to Euros (€), using an exchange rate of ¥100=€120.

### Sensitivity analyses

Sensitivity analysis was used to handle parameter uncertainty. It is possible that differences exist in chemotherapy drug dosage administered to Euro-American and Japanese patients. Japanese individuals generally weigh less and have a smaller body surface area relative to Euro-Americans. As such, a sensitivity analysis was performed for dosage. Notably, unadjusted dosage was used in the base-case analysis. Impact of hospitalisation cost for AEs was also analysed because patient-level data were unavailable. Uncertainty of the incremental cost-effectiveness ratio was evaluated, based on the bootstrap method (in which bootstrap resampling was repeated 10 000 times). In addition, we calculated medical costs for National Health Service (NHS) in the UK health-care setting. This value incorporated anticancer drug costs (Pandor *et al*, 2006; Joint Formulary Committee, 2007), administration costs (£109 per cycle), infusion pump costs (£62 per cycle), pharmacy costs (£38 per i.v.), hospital admission (£258 per day), clinician consultations (£80 per cycle), and cost of diagnostic tests (£65 per chemotherapy) (Tappenden *et al*, 2007).

## Results

### Effectiveness

Table 2 displays estimated utility scores for AEs, chemotherapy regimens without AEs, and the 95% CIs. As a chemotherapy regimen for the general population, XELOX was generally preferred over FOLFOX4. Adverse event decreased utility scores by about 0.1–0.2. These utility scores were used to calculate mean QAPFSD of each patient group.

Results of the cost-effectiveness analysis are displayed in Table 3. Incremental effectiveness of XELOX was significantly larger than 0 for both first-line and second-line therapy. Incremental effectiveness of first-line XELOX for MCRC patients was 10.5 QAPFSD, whereas incremental effectiveness of second-line XELOX for MCRC patients was 11.3 QAPFSD. The difference in PFSD was −9.3 PFSD for first-line MCRC patients and 2.2 PFSD for second-line MCRC patients. The PFSD difference between XELOX and FOLFOX4 was not statistically significant.

### Costs

Capecitabine plus oxaliplatin (XELOX) was proven to significantly reduce treatment costs by €3000 (JPY 360 000) in first-line treatment and €2300 (JPY 270 000) in second-line treatment, as compared with FOLFOX4 involving branded l-LV. Even if the comparator regimen was changed to FOLFOX4 involving generic l-LV, XELOX decreased treatment costs by €1800 (JPY 220 000) in first-line therapy and €1500 (JPY 180 000) in second-line therapy.

Detailed costs are shown in Table 4. Cost of chemotherapy was the major component in both groups analysed. Difference in chemotherapy costs and outpatient chemotherapy fee is the factor that had the most influence on the incremental costs of XELOX.

### Cost-effectiveness

Both first-line and second-line treatments were proven to be ‘dominant’ (Table 3). These results mean that costs of XELOX were lower, and more QALY could be gained by use of XELOX.

### Sensitivity analysis

Lower doses of XELOX resulted in lower incremental costs of XELOX. If the dose of first-line chemotherapy was reduced by 90%, costs could be reduced by €3300 (JPY 400 000), as compared with FOLFOX4 involving branded l-LV; or by €2300 (JPY 270 000), as compared with FOLFOX4 involving generic l-LV. If the dose of second-line chemotherapy was reduced by 90%, costs could be reduced by €3800 (JPY 450 000), as compared with FOLFOX involving branded l-LV; or by €2800 (JPY 340 000), as compared with FOLFOX4 involving generic l-LV.

**Figure 2:**
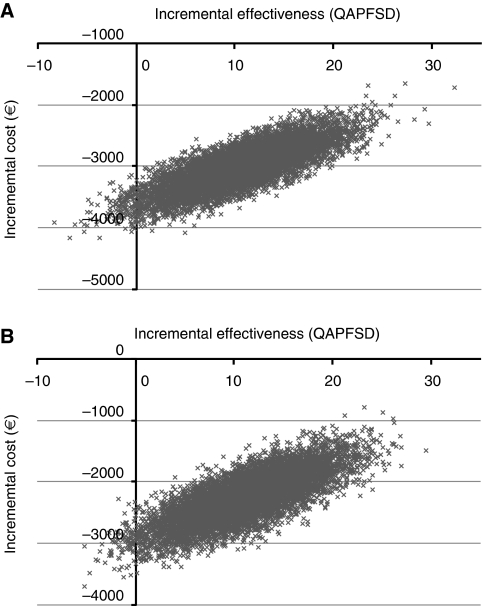
The distribution of incremental effectiveness and cost. (**A**) first-line therapy; (**B**) second-line therapy.

The mean length of hospitalisation per patient was 1.14 days (XELOX) and 0.77 days (FOLFOX4) in the NO16966 trial (Scheithauer *et al*, 2007). No data were available on actual resource consumption. Even if we assumed that all patients were treated in an intensive care unit, resulting in a daily cost of €730 (JPY 87 600) per day), the difference in cost was €270 (JPY 32 000). Thus, the difference in length and type of stay had minimal, if any, influence on total costs. More than 95% resampling data on incremental effectiveness and costs were distributed in the ‘dominant’ quadrant, which was shown in Figure 2.

In the UK health-care setting, costs of FOLFOX4 and XELOX as first-line therapy were estimated to be £23 600 (£14 000; chemotherapy drug costs) and £16 100 (£12 300; chemotherapy drug costs), respectively. Capecitabine plus oxaliplatin decreased treatment costs by £7600. In second-line therapy, costs of FOLFOX4 were £13 600 (£8600; chemotherapy drug costs), whereas XELOX cost £9700 (£7600; chemotherapy drug costs). The difference in cost was estimated to be £3900. In the UK health-care setting, XELOX was also superior to FOLFOX4 with regard to cost.

### Budget impact

Our findings have important implications for oncology expenditures. An estimated 40 000 patients are newly diagnosed with MCRC in Japan each year. Assuming that use of XELOX decreased the medical costs for an MCRC patient by €3000 (JPY 360 000) and FOLFOX4 is administered to at least one-fourth or half of newly diagnosed MCRC patients, an annual savings of approximately €30 million (JPY 3.6 billion) or €60 million (JPY 7.2 billion) could be possible in Japan. In the United Kingdom, the number of patients is estimated to be 12 665 (Hind *et al*, 2008). On the basis of our assumption that first-line XELOX decreased medical costs for NHS by £7600, XELOX helps to reduce the total budget by a maximum of £96 million.

## Discussion

Because the FOLFOX4 regimen is currently administered to many newly diagnosed MCRC patients, a comparison of cost-effectiveness of XELOX and FOLFOX4 is important. Here, we showed that first-line and second-line XELOX chemotherapy for MCRC was ‘dominant’ (i.e., showed better efficacy and resulted in lower treatment costs), compared with FOLFOX4. These results were not influenced by the sensitivity analysis. Our results are also statistically robust because predicted values by linear or nonlinear regression were not used.

Large sample size may have led to statistically significant differences, but effect size was not very large. We are unsure of the clinical significance of these differences, and it may therefore be more appropriate to conclude that at minimum, XELOX produced no fewer QALYs than did FOLFOX4. If the advantage of XELOX in terms of personal preference is not taken into account, PFSD values for XELOX and FOLFOX4 are not significantly different. Given the non-inferiority of XELOX observed in both NO16966 and NO16967 trials, XELOX is superior in cost and non-inferior in PFSD to FOLFOX4.

Very few data are available for an economic evaluation, which compares oral chemotherapy with intravenous chemotherapy for MCRC patients. In the United States, direct medical costs for MCRC patients in the same NO16966 trial were estimated at US$ 44 500 for XELOX and US$ 45 800 for FOLFOX4 (Garrison *et al*, 2007; Scheithauer *et al*, 2007; Chu and Cartwright, 2008). In the United Kingdom, a cost-minimisation analysis showed the benefit of oral chemotherapy by showing that treatment costs for a 12-week course of capecitabine (£2132) were lower than the costs for the Mayo regimen (£3593), de Gramont regimen (£6255), and modified de Gramont regimen (£3485) schedules over the same treatment period (Ward *et al*, 2006). A Canadian study (Maroun *et al*, 2003) reported similar findings in which oral uracil-tegafur (UFT) regimen (UFT plus oral LV) saved 3221 Canadian dollars per treatment compared with intravenous 5-FU plus oral LV. On the basis of these results, oral chemotherapies result in lower costs than intravenous therapies. This is consistent with our present results.

Several limitations and strengths of our study should be noted. Our analysis did not consider indirect costs, such as work loss resulting from chemotherapy. Capecitabine plus oxaliplatin does not require continuous infusion of 5-FU, and administration of XELOX chemotherapy is less frequent than that of FOLFOX4. If we include direct non-medical cost (such as costs of transportation of patients to clinics) and indirect costs from the societal perspective, incremental costs of XELOX would be even lower than the current analysis from the payer’s perspective.

Although our study showed benefits of effectiveness of XELOX, clinical trials proved that XELOX was non-inferior, rather than superior, to FOLFOX4. This is mainly because a preference study showed that general people preferred oral capecitabine, rather than intravenous, chemotherapy. We think this is a reasonable result. Moreover, the profile of grade 3/4 AEs was not different, except that hand–foot syndrome occurred more frequently in the XELOX group. However, utility scores were measured by Web-based survey, and respondents were sampled from Internet panels, rather than by random sampling from the population. Although we recruited respondents stratified by age and gender, it is possible that the characteristics of respondents or responses to the questions were different than from a survey conducted among the general population. This is also a limitation of this study.

We believe that XELOX effectiveness does not vary significantly between Japanese and Euro-American patients. In the field of oncology, recent methods of chemotherapy administration are universally adopted. This is especially true for clinical trials in which treatment methods such as chemotherapy regimen or diagnostic imaging frequency are standardised by the protocols. Therefore, a difference in resource use is also unlikely. The dose of anticancer drugs administered, however, are lower in Japan, due to the smaller body surface area of patients. Sensitivity analysis on the dose of anticancer drugs showed that incremental costs were lower if dose was reduced. The difference in costs between XELOX and FOLFOX4 may expand in the Japanese setting. Furthermore, the lack of a comparison with other oral chemotherapy drugs, such as (UFT) or tegafur-gimestat-otastat potassium (TS-1), which are widely used in Japan, is another limitation of our study.

According to our analysis, first-line and second-line use of XELOX chemotherapy was superior to FOLFOX4 in terms of effectiveness and costs. Although FOLFOX is a standard regimen for MCRC patients, we recommend the use of the XELOX regimen as a treatment option for MCRC patients.

## Figures and Tables

**Table 1:** Baseline patient characteristics

	**NO16966 trial**		**NO16967 trial**	
**Chemotherapy regimen**	**FOLFOX4**	**XELOX**	**FOLFOX4**	**XELOX**
*Patient characteristics*				
Number of patients	649	655	308	311
Male gender (%)	57.9	59.8	60.7	62.1
				
*Race*				
Caucasian (%)	81.7	82.2	81.8	81.7
Black (%)	1.7	2.0	1.9	3.2
Other (%)	16.6	15.7	16.2	15.1
				
Age (years)	59.7	59.7	59.7	60.7
Weight (kg)	72.6	72.6	77.5	75.5
				
*ECOG performance*				
0 (%)	56.1	55.0	46.4	48.2
1 (%)	43.8	45.0	46.8	44.3
2 (%)	—	—	6.8	7.4

**Table 2:** Utility scores for metastatic colorectal cancer

	***N***	**Utility score**	**95% CI**
(a) *Chemotherapy*			
XELOX without adverse events	191	0.59	0.55–0.64
FOLFOX without adverse events	183	0.53	0.49–0.57
			
(b) *Adverse events*			
Febrile neutropenia	175	0.39	0.36–0.42
Nausea/vomiting	192	0.38	0.35–0.42
Diarrhoea	188	0.42	0.39–0.45
Hand–foot syndrome	174	0.39	0.36–0.42
Fatigue	185	0.45	0.41–0.48
Peripheral neuropathy	176	0.45	0.41–0.48
Stomatitis	202	0.42	0.39–0.45

CI=confidence interval.

**Table 3:** Results of the cost-effectiveness analysis

**(a) Cost**				
	**First-line therapy**		**Second-line therapy**	
**Chemotherapy regimen**	**Estimated cost (€)**	**95% CI**	**Estimated cost (€)**	**95% CI**
(1) XELOX	18 300	17 900–18 700	12 600	12 200–13 100
(2) FOLFOX (branded l-LV)	21 300	20 800–21 800	14 900	14 300–15 500
(3) FOLFOX (generic l-LV)	20 200	19 700–20 600	14 100	13 600–14 700
(1)–(2)	−3000	−3600 to 2400	−2300	−1600 to 3000
(1)–(3)	−1900	−2500 to 1200	−1500	−2200 to 800
				
**(b) Effectiveness (QAPFSD)**				
	**First-line therapy**		**Second-line therapy**	
**Chemotherapy regimen**	**Estimated Effectiveness (QAPFSD)**	**95% CI**	**Estimated Effectiveness (QAPFSD)**	**95% CI**
(1) XELOX	149.1	141.9–156.2	97.8	91.2–105.3
(2) FOLFOX	138.5	132.3–144.6	86.5	80.5–92.6
(1)–(2)	10.5	1.0–20.2	11.3	2.2–20.8
				
**(c) Effectiveness (PFSD)**				
	**First-line therapy**		**Second-line therapy**	
**Chemotherapy regimen**	**Estimated Effectiveness (PFSD)**	**95% CI**	**Estimated Effectiveness (PFSD)**	**95% CI**
(1) XELOX	253.4	241.2–265.9	165.8	153.9–178.3
(2) FOLFOX	262.7	251.0–274.6	163.6	152.2–175.6
(1)–(2)	−9.3	−26.7 to 7.6	2.2	−14.4 to 19.2

CI=confidence interval; LV=leucovorin; PFSD=progression-free survival days; QAPFSD=quality-adjusted progression-free survival days.

**Table 4:** Detailed mean costs per patient until progression of disease

	**Costs (€) of first-line chemotherapy**		**Costs (€) of second-line chemotherapy**	
**Chemotherapy regimen**	**FOLFOX4**	**XELOX**	**FOLFOX4**	**XELOX**
Oxaliplatin	12 200	11 500	8800	8300
l-LV (branded)	3800		2600	
l-LV (generic)	2700		1800	
5-FU	600		400	
Capecitabine	—	3500	—	2300
Subtotal (1) Chemotherapy costs	16 600	15 000	11 800	10 600
Subtotal (1)’ Chemotherapy costs (involving generic l-LV)	15 500		11 000	
				
5-HT3 antagonists	1050	600	800	410
Corticosteroids	70	40	50	30
Other medications	4	2	8	1
Subtotal (2) Pretreatment costs for chemotherapy	1100	600	900	440
				
Outpatient chemotherapy fee	1400	420	920	330
Blood test	750	750	420	420
Imaging diagnosis	1400	1400	750	830
Pharmacy fee		80		60
Subtotal (3) Outpatient chemotherapy costs	3600	2700	2100	1600
Subtotal (4) Medication costs for AEs	100	40	100	30
				
Total costs	21 300	18 300	14 900	12 600
Total costs (involving generic l-LV)	20 200		14 100	

AEs=adverse events; 5-FU=5-fluorouracil; LV=leucovorin.
